# Disappearing Colorectal Liver Metastases: Should the Treatment Sequence Be Reconsidered for Selected Lesions?

**DOI:** 10.3390/cancers18182994

**Published:** 2026-09-16

**Authors:** Alessandro Giacomoni, Michele Paterno, Paolo Aseni, Antonio Benedetti, Pietro Calcagno, Marco Solcia

**Affiliations:** 1 Hepatobiliary Surgical and Abdominal Organ Transplantation Unit, Fondazione IRCCS San Matteo, 27100 Pavia, Italy; 2 Minimally Invasive Surgical Oncology Unit, ASST Grande Ospedale Metropolitano Niguarda Hospital, 20162 Milan, Italy; michele.paterno.94@gmail.com (M.P.); antonio.benedetti@ospedaleniguarda.it (A.B.); pietro.calcagno@ospedaleniguarda.it (P.C.); 3 Department of Emergency Medicine, ASST Grande Ospedale Metropolitano Niguarda Hospital, 20162 Milan, Italy; paolo.aseni@ospedaleniguarda.it; 4 Interventional Radiology Unit, ASST Grande Ospedale Metropolitano Niguarda Hospital, 20162 Milan, Italy; marco.solcia@ospedaleniguarda.it

**Keywords:** colorectal cancer, liver metastases, disappearing liver metastases, thermal ablation, treatment sequencing, parenchyma-sparing treatment, fiducial markers, hepatobiliary MRI

## Abstract

Disappearing liver metastases (DLMs) remain a challenging problem in the management of colorectal liver metastases (CRLMs). Although modern systemic therapy may produce complete radiological responses, radiological disappearance does not reliably indicate complete pathological response. Conversely, mandatory treatment of every original metastatic site may cause unnecessary parenchymal sacrifice when a lesion is no longer identifiable on hepatobiliary contrast-enhanced magnetic resonance imaging or intraoperative ultrasonography. Current strategies—including treatment of the presumed site, selective pre-treatment fiducial marking where feasible and considered appropriate, and structured surveillance—therefore involve important trade-offs. We propose a hypothesis-generating, lesion-specific strategy for a narrowly selected subgroup: thermal ablation before systemic therapy of one or a few small CRLM, preferably no larger than 2 cm, considered at higher anticipated risk of post-treatment loss of localization. Selection should distinguish the probability of radiological disappearance from its clinical consequences. Candidate lesions should be clearly and unambiguously identifiable on high-quality baseline imaging, safely accessible for ablation with a margin greater than 5 mm and ideally approximately 10 mm, and located outside any planned resection field, particularly when their depth or anatomical position would make empirical treatment difficult after disappearance. Recent randomized evidence supports thermal ablation as definitive local therapy for appropriately selected small CRLM but does not validate the proposed pre-systemic treatment sequence. This approach should not delay urgently required systemic therapy, compromise biological selection, or be used indiscriminately in high-volume, rapidly progressive, uncontrolled extrahepatic, or technically unsuitable disease. It is also generally unnecessary when the lesion is encompassed by a planned resection or total hepatectomy within a liver transplantation pathway. Upfront ablation may secure local control while the target remains visible, preserve liver parenchyma, and avoid premature interruption of systemic therapy or acceleration of surgery solely because a responding lesion is becoming radiologically occult. However, it may cause overtreatment and procedural morbidity and remains unsupported by prospective comparative evidence. A multicentre feasibility study, followed by prospective comparison with contemporary standard sequencing and, where feasible and considered appropriate, fiducial-based localization, is required to evaluate this strategy.

## Simple Summary

Modern drug treatment can make small colorectal liver metastases disappear on scans, even though living cancer cells may remain. Once no longer visible, these lesions can be difficult to remove or destroy accurately. This article examines whether, in carefully selected patients, one or a few small metastases at high risk of becoming untraceable could be destroyed by heat before systemic therapy. Candidates would require clearly visible lesions, safe access, an adequate treatment margin, and no urgent need to start systemic therapy. This lesion-specific approach could preserve healthy liver tissue and avoid interrupting effective drug treatment because a target is disappearing. However, it could also expose patients to an unnecessary procedure if drugs would have eradicated the lesion completely. This proposal is not a new standard of care, but a research hypothesis that should be tested prospectively against current strategies, including marker placement and careful imaging surveillance.

## 1. The Clinical Problem of Disappearing Liver Metastases

The increasing efficacy of systemic therapy has changed the therapeutic landscape of colorectal liver metastases (CRLMs). Lesions that are clearly measurable on baseline imaging may become undetectable after chemotherapy, giving rise to the phenomenon of disappearing liver metastases (DLMs). This finding may reflect favourable tumour biology and a major treatment response; however, complete radiological response is not synonymous with complete pathological response [1,2,3,4]. A lesion that is no longer visible on imaging may still contain viable tumour cells, whereas another lesion may indeed have undergone durable sterilization. This uncertainty is the source of the practical dilemma.

The problem is clinically relevant because treatment decisions in CRLMs are increasingly lesion-specific. A patient may present with multiple hepatic metastases showing heterogeneous behaviour during therapy: some lesions remain visible, some shrink but persist, and one or more may disappear radiologically. The clinician must therefore decide not only whether the patient is responding overall but whether each original site still requires local treatment.

The broader debate on treatment sequencing provides relevant context. Although adjuvant chemotherapy improves overall survival in stage III colon cancer, randomized trials in patients with resectable CRLMs have generally shown gains in progression-related endpoints without a consistent overall survival advantage from routine perioperative or adjuvant systemic therapy. Accordingly, a growing, although not uniform, body of literature has questioned an undifferentiated chemotherapy-first default in patients with clearly resectable CRLMs, favouring sequencing based on anatomy, tumour biology, and individual risk [5,6]. This literature also highlights potential costs of induction therapy, including chemotherapy-associated hepatic injury, increased perioperative morbidity, distortion of radiological appearances, and progression during treatment that may close a window for curative local therapy. These considerations do not establish a role for routine upfront ablation, but they do strengthen the rationale for asking whether a clearly visible, safely treatable lesion should invariably be exposed to the risk of radiological disappearance before local control is secured.

DLMs should also be defined carefully. A lesion should not be labelled as disappearing merely because it is no longer visible on computed tomography. Contemporary assessment requires high-quality baseline imaging and restaging with hepatobiliary contrast-enhanced magnetic resonance imaging (MRI), ideally reviewed side by side by an experienced hepatobiliary radiologist [4,7]. Contrast-enhanced intraoperative ultrasonography (CE-IOUS) may further improve intraoperative detection, yet radiological disappearance still does not establish complete non-viability with certainty [4,8].

Contemporary series indicate that at least one DLM occurs in approximately 7–48% of patients receiving systemic therapy (median about 19%). Across heterogeneous imaging protocols, complete pathological or durable clinical response at a disappearing site has ranged from approximately 17% to 85%. Importantly, when a lesion remains occult on both hepatobiliary MRI and intraoperative ultrasonography, particularly contrast-enhanced intraoperative ultrasonography, the probability of a complete response appears substantially higher, approaching 75–94% in selected modern series. Historical reports nevertheless describe site-specific recurrence in approximately 33–74% of untreated DLMs, although these estimates predate uniform use of contemporary hepatobiliary MRI and CE-IOUS and should be interpreted cautiously [7,8].

## 2. Why Current Strategies Remain Imperfect

Current sequencing is tailored to resectability and tumour biology. Patients with limited, clearly resectable liver-only disease may proceed directly to parenchyma-sparing resection, with or without perioperative chemotherapy; borderline resectable or initially unresectable disease generally receives conversion therapy followed by reassessment. Multifocal bilobar disease may require staged hepatectomy, portal-vein embolization, or combinations of limited resection and ablation to achieve complete treatment while preserving an adequate future liver remnant [9]. If a lesion disappears during this pathway, options include treatment of its presumed site using baseline imaging and intraoperative ultrasonography, pre-treatment fiducial marking, or structured surveillance with salvage treatment [10]. Each option balances oncological completeness against procedural morbidity and loss of normal parenchyma [7,10,11]. Treating every original metastatic site is oncologically intuitive because residual viable cells may remain despite radiological disappearance. When the presumed site lies within a planned hepatectomy, this objective may be achieved without materially changing the procedure.

The challenge emerges when a small, deep lesion lies outside the intended field of resection. Once such a lesion disappears, empirical treatment based on pre-chemotherapy imaging and indirect anatomical landmarks may require unnecessary sacrifice of normal parenchyma, may miss the true target, or may expose the patient to procedural risk without clear benefit. Active surveillance preserves liver tissue and avoids immediate overtreatment, while allowing salvage intervention if the lesion reappears. However, it accepts a meaningful risk of site-specific recurrence. The available evidence is largely retrospective and limited by selection bias, heterogeneous imaging protocols, non-uniform definitions of DLMs, and differences in systemic regimens [9,10,11]. Although unresected DLMs appear to recur more often at the original site, a clear overall survival benefit from mandatory treatment of every DLM has not been consistently demonstrated.

Pre-treatment fiducial placement represents a rational intermediate strategy, but its practical limitations should not be underestimated. By marking selected lesions before chemotherapy, it may preserve anatomical localization without immediately destroying tumour tissue and facilitate delayed resection or ablation after systemic treatment. However, this approach is not universally available, as it depends on access to an experienced interventional radiology team capable of placing the marker accurately and safely. Fiducial placement is itself invasive and may be complicated by bleeding, migration, or inaccurate positioning. In these cases if surgical retrieval of the marker is required, it generally entails excision of a small amount of surrounding liver parenchyma; depending on the depth of the marker and its relationship to major vascular or biliary structures, this may be technically demanding and may partly undermine the intended parenchyma-sparing strategy. Fiducial marking should therefore be considered cautiously, after multidisciplinary discussion, and only when its expected value for subsequent lesion localization clearly outweighs these procedural and anatomical limitations. Conceptually, fiducial marking and upfront ablation address the same anticipated localization problem through different trade-offs: marking preserves treatment optionality and avoids definitive local therapy before biological assessment but requires an invasive localization procedure and may still be affected by migration or imperfect targeting; upfront ablation secures immediate local control while the lesion is visible but accepts procedural morbidity and the possibility of overtreatment.

## 3. Why Thermal Ablation Changes the Discussion

Thermal ablation has evolved considerably in the treatment of CRLMs. It is no longer viewed exclusively as a salvage option for unresectable disease but rather as a credible local treatment in selected patients with limited tumour burden and technically favourable lesions. Recent prospective evidence, including the COLLISION trial, has strengthened the position of ablation as a potentially definitive local therapy for small, resectable and ablatable metastases, particularly when performed in experienced centres and with adequate margins. The proposed strategy may be performed using either microwave ablation (MWA) or radiofrequency ablation (RFA). Although MWA may offer shorter treatment times and reduced susceptibility to heat-sink effects in selected anatomical settings, no evidence specific to lesions at risk of becoming DLMs supports a universal preference for one modality. The technique should therefore be selected according to lesion anatomy, the required ablation volume, proximity to vessels and vulnerable structures, procedural route, available technology, and operator expertise. The decisive requirement is safe and complete treatment with an adequate ablation margin, rather than the energy source itself [12].

The international phase III COLLISION trial provides the strongest context for this argument, although it did not investigate DLM or treatment timing. Across 14 centres, adults with up to ten resectable and ablatable CRLMs measuring 3 cm or less, no extrahepatic disease, and Eastern Cooperative Oncology Group performance status 0–2 were randomized to ablation or resection of all target lesions. At the prespecified interim analysis, 296 eligible patients had been followed for a median of 28.9 months. Median overall survival was not reached in either group (hazard ratio 1.05, 95% confidence interval 0.69–1.58), local control was non-inferior, and predefined stopping criteria were met. Ablation caused fewer adverse events (19% vs. 46%), fewer serious adverse events (7% vs. 20%), and no treatment-related deaths versus three after resection. These results do not validate pre-chemotherapy ablation of lesions considered at higher anticipated risk of post-treatment loss of localization, but they support examining sequence as a separate clinical question [13].

The evidentiary distinction is essential: COLLISION supports thermal ablation as definitive local therapy for appropriately selected small CRLMs, but it did not study DLM, prediction of disappearance, or ablation before systemic therapy. It therefore supports the local-treatment modality, not the proposed treatment sequence, which remains untested and hypothesis-generating.

This evolution matters because it changes how one might think about DLMs. If thermal ablation can offer durable local control in carefully selected lesions, then the timing of ablation becomes a legitimate conceptual question. The issue is not merely whether ablation is technically feasible after chemotherapy but whether certain lesions should be treated before systemic therapy precisely because they may later become difficult to identify or target conservatively.

This is especially relevant for small, deeply located lesions that are clearly visible before treatment but are considered at higher anticipated risk of post-treatment loss of localization after a strong systemic response. Once they vanish radiologically, management becomes reactive and uncertain. Upfront ablation would instead secure local treatment while the target is still visible and technically accessible.

## 4. A Selective Alternative Sequence

Here, upfront therapy means lesion-directed ablation performed after complete staging and multidisciplinary review but before the first planned cycle of systemic therapy. It does not mean ablating every hepatic deposit or subordinating chemotherapy in aggressive, extensive, or symptomatic disease. It is instead a pre-emptive strategy for one or a few technically favourable lesions whose disappearance could frustrate accurate, parenchyma-sparing treatment. The interval to chemotherapy should be predefined and short; the procedure is acceptable only when expected morbidity is low and systemic treatment will not be materially delayed.

We therefore propose consideration of upfront thermal ablation for a narrow and carefully selected subgroup of patients who are already candidates for curative-intent multimodality treatment. The target lesion should be small, preferably no larger than 2 cm, clearly visible on high-quality baseline imaging, technically accessible for safe ablation, and located outside a planned resection field.

It should also be considered at higher anticipated risk of becoming difficult to localize or treat conservatively after systemic therapy. Throughout the proposed framework, candidate lesions are defined consistently as preferably no larger than 2 cm. This is a pragmatic, hypothesis-generating criterion rather than a prospectively validated absolute cutoff. Complete tumour coverage should be achievable with a circumferential ablation margin greater than 5 mm; whenever anatomically feasible and safe, a margin of approximately 10 mm should be pursued because it is associated with more favourable local tumour control [14].

The preferred 2 cm threshold is deliberately more restrictive than the 3 cm upper limit used in COLLISION. It is not proposed as a general limit for CRLM ablation; rather, it enriches the framework for lesions more likely to become radiologically occult while also favouring reproducible complete coverage and an adequate safety margin. Lesions measuring 2–3 cm may remain appropriate for ablation in standard practice, but their inclusion in this pre-systemic hypothesis should require particularly favourable anatomy and careful prospective documentation. It should also be judged likely to become difficult to localize or difficult to treat conservatively after chemotherapy.

**Figure 1:**
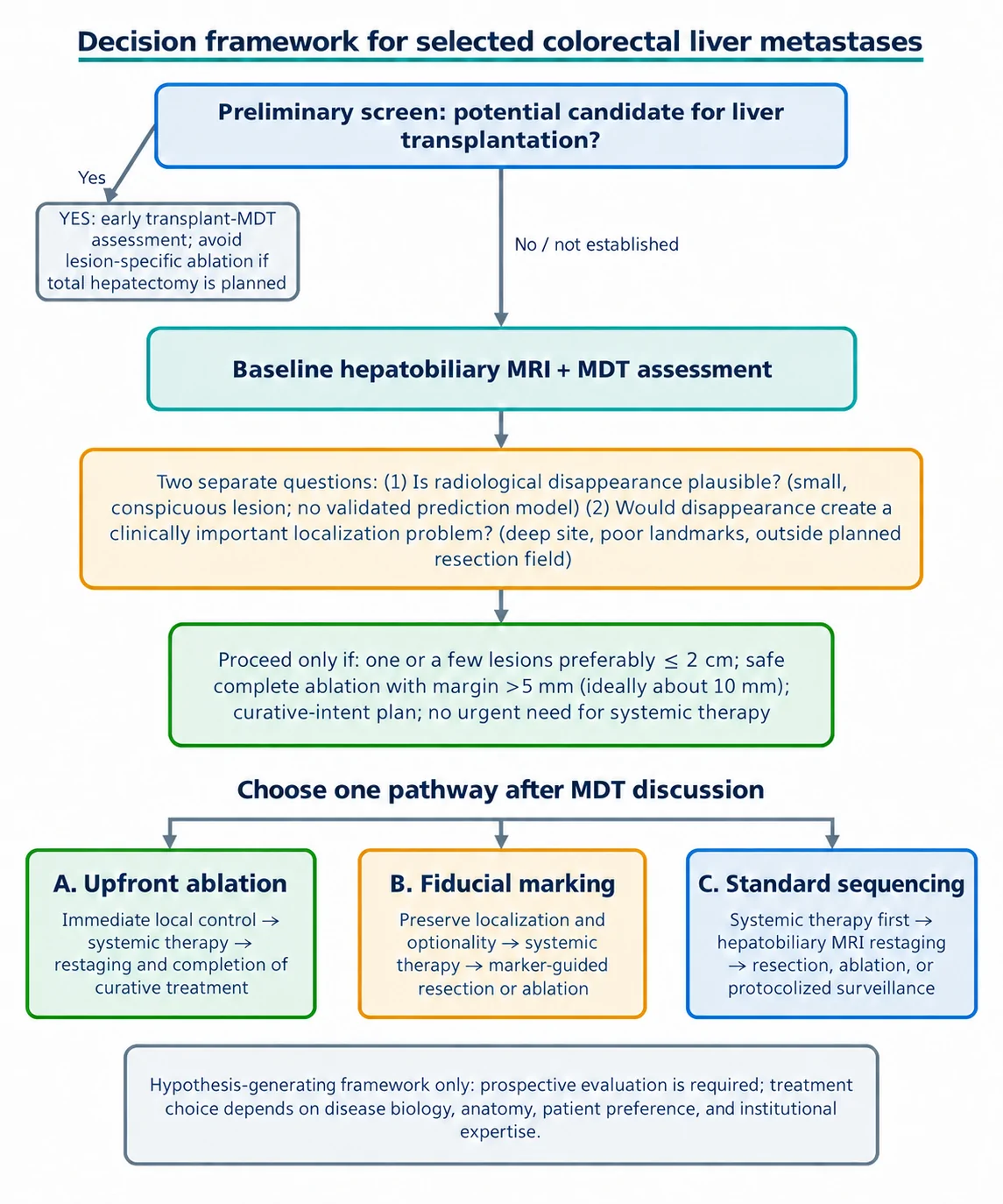
Proposed decision framework for considering upfront thermal ablation in a narrowly selected subgroup of colorectal liver metastases at higher anticipated risk of post-treatment loss of localization. The strategy is restricted to lesions that are small, technically accessible, outside a planned resection field, and unlikely to be safely or conservatively managed if radiological disappearance occurs.

This proposal should be considered only within a multidisciplinary framework involving hepatobiliary surgery, medical oncology, interventional radiology, diagnostic radiology, and, when appropriate, colorectal surgery. The rationale should be prospective and explicit: the lesion is not being ablated upfront because all metastases should be treated immediately but because delaying local treatment may convert a visible, straightforward target into a clinically ambiguous and technically problematic one. A proposed decision framework for this selective strategy is shown in Figure 1.

The algorithm displays the principal eligibility criteria directly, including preferred lesion size ≤2 cm, clear baseline visibility, higher anticipated risk of loss of localization, location outside the planned resection field, an achievable margin >5 mm (ideally approximately 10 mm), absence of a need for urgent systemic therapy, and preliminary assessment of potential liver transplant candidacy. The revised algorithm also separates the probability of disappearance from the consequences of loss of localization and presents three alternative sequences: upfront ablation, fiducial marking, and standard systemic-therapy-first management.

In practical terms, the decision would most likely arise in patients for whom systemic neoadjuvant therapy is planned anyway, but in whom one or a few lesions create a disproportionate technical concern because of their size, depth, or anticipated post-treatment invisibility. The purpose of upfront ablation would not be to replace the global treatment plan but to secure control of a lesion that may otherwise become a source of uncertainty during subsequent surgery or follow-up.

Several exclusions are important. This strategy should not delay urgently needed preoperative systemic therapy. It should not be used when lesions are already included in a planned parenchyma-sparing resection, when the ablation margin is likely to be inadequate, when the lesion is close to major bile ducts or other vulnerable central structures, or when the diagnosis remains uncertain. Histological confirmation and molecular profiling should already be available from the primary tumour or another suitable site whenever these data are required to guide systemic treatment.

## 5. Potential Advantages, Risks, and Competing Strategies

The principal theoretical advantage of upfront ablation is simple: local treatment is completed before the lesion is lost as an anatomical target. This may reduce later uncertainty, avoid empirical parenchymal sacrifice, preserve future liver-directed options, and simplify the management of lesions that would otherwise become radiologically occult. It may be particularly attractive when disappearance would transform a straightforward percutaneous target into an unlocalizable deep site.

However, the counterarguments are substantial and should not be minimized. Upfront ablation may overtreat lesions that would have achieved durable complete response with systemic therapy alone. It introduces procedural morbidity, may delay chemotherapy, may complicate subsequent imaging interpretation, and may eliminate tissue that could otherwise contribute to pathological or translational assessment. In addition, excellent local control of one lesion does not alter the systemic biological risk carried by the patient.

Systemic therapy also functions as a test of tumour biology. Rapid hepatic or extrahepatic progression during initial treatment may identify patients unlikely to benefit from aggressive local therapy; in retrospect, upfront ablation in such patients would have been an unnecessary invasive procedure. The value of preserving this biological-selection interval should therefore be weighed explicitly against the anatomical risk that a target lesion will disappear and become difficult to localize.

This argument should not be interpreted as minimizing the value of systemic therapy. Preoperative treatment remains appropriate when downsizing is required, when bilobar or technically complex disease calls for a staged strategy, when the future liver remnant is marginal, when adverse biological features suggest a high risk of occult systemic disease, or when synchronous primary and hepatic disease requires coordinated sequencing [12]. Our proposal is therefore lesion-specific and embedded within a multimodality plan; it is not a general surgery-first or ablation-first policy for all patients with resectable CRLMs [15,16].

The main potential advantages and drawbacks of this approach are summarized in Table 1. The relevant comparison is therefore not ablation versus no treatment but rather a set of competing precision strategies: immediate ablation, pre-treatment fiducial marking followed by delayed local treatment, planned resection, or active surveillance with clearly defined salvage criteria. The best option is likely to depend on lesion anatomy, total disease burden, urgency of systemic therapy, technical expertise, institutional resources, and patient preference.

## 6. Toward More Explicit Selection Criteria

If this concept is to be clinically useful, selection must be more explicit than lesions preferably no larger than 2 cm and at higher anticipated risk of post-treatment loss of localization. Candidate lesions would probably share several features: small size, deep parenchymal location, clear visibility and unambiguous localization on high-quality baseline imaging, technical suitability for percutaneous or laparoscopic ablation, and a location outside any planned resection field. They may also belong to patients with otherwise controllable disease, in whom liver preservation matters because repeat intervention may later be required.

Conversely, lesions already included in a limited anatomic or parenchyma-sparing resection are poor candidates for this strategy, since upfront ablation would add complexity without clear gain. Likewise, lesions adjacent to central bile ducts, lesions with uncertain diagnosis, or lesions in patients with rapidly progressive extrahepatic disease are not suitable. In this sense, the proposal is intentionally narrow.

Other potentially relevant selection variables might include the quality and reproducibility of baseline imaging, centre-specific ablation expertise, and the anticipated need for future repeat local treatment. These factors are not yet validated as formal criteria, but they do illustrate why the strategy should be developed through prospective refinement rather than immediate routine adoption.

Three complementary domains may provide a more reproducible basis for selection. For practical purposes, eligibility can be organized into three complementary domains: technical ablatability, anticipated risk of post-treatment loss of localization, and the clinical urgency of systemic therapy. These domains should be documented separately at the multidisciplinary meeting. They are intended to structure, rather than replace, expert judgment, because no prospectively validated model currently predicts the disappearance of an individual CRLM with sufficient accuracy to determine treatment. Two distinct dimensions should be assessed separately. The first is the probability of radiological disappearance during systemic therapy, for which small lesion size and treatment-related response patterns provide only non-validated associations. The second is the clinical consequence if disappearance occurs, determined principally by anatomy: a deep lesion, one lacking durable landmarks, or one outside the planned resection field may become difficult to treat without empirical parenchymal sacrifice. A lesion should be considered for the proposed strategy only when both dimensions are clinically relevant and the lesion is safely ablatable.

Technical ablatability. The target should preferably measure no more than 2 cm, be unequivocally identifiable on baseline and procedural imaging, and have a safe percutaneous or operative access route. Complete tumour coverage should be achievable with a minimum ablation margin greater than 5 mm and, whenever anatomy permits, approximately 10 mm, because smaller margins are associated with a substantially greater risk of local tumour progression [14]. Lesions abutting central bile ducts or other vulnerable structures, lesions affected by a major heat-sink limitation from an adjacent large vessel, and lesions for which no safe applicator trajectory exists should be excluded unless protective or alternative technical measures can reliably overcome these limitations [12]. A lesion already encompassed within a planned anatomic or parenchyma-sparing resection is not an appropriate target for pre-treatment ablation.

Anticipated risk of becoming difficult to localize.

Available evidence identifies associations rather than a validated prediction rule. DLMs have been reported more frequently among lesions smaller than 2 cm and in patients with synchronous metastases, at least three CRLMs, exposure to a greater number of treatment cycles, and oxaliplatin-containing systemic therapy [17,18,19]. The likelihood of complete radiological response may also increase with highly active systemic and molecularly targeted regimens. A very small lesion that is clearly visible at baseline but has limited conspicuity or few reproducible anatomical landmarks may therefore be considered at higher risk of becoming untraceable after a major response. Early, marked dimensional reduction on interval imaging may reinforce this concern, but it becomes available only after treatment has begun and cannot independently justify a strictly pre-treatment intervention. Accordingly, the proposed indication should be described as a higher anticipated risk of post-treatment loss of localization, not as a confidently predictable DLM.

Clinical and biological variables. CEA, CA 19-9, primary tumour T and N stage, sidedness, histological features, molecular profile, disease-free interval, and synchronous versus metachronous presentation contribute to assessment of overall tumour biology. Elevated CEA and CA 19-9 have prognostic value in CRLMs, particularly when interpreted together with lesion number and size [17]. However, neither marker has a validated threshold for predicting whether a specific metastasis will disappear, and a decline in tumour markers during treatment occurs too late to guide a purely upfront procedure. Primary tumour stage and location have likewise shown heterogeneous associations with treatment response and should not be used as stand-alone lesion-selection criteria. These variables should instead help determine whether the patient remains an appropriate candidate for curative-intent multimodality therapy and whether systemic control should take priority.

Urgency of systemic therapy. Patients should not undergo pre-treatment ablation when even a short procedural interval could compromise systemic control. Exclusion should be considered in rapidly progressive or high-volume disease, borderline-resectable or unresectable disease requiring prompt conversion therapy, clinically significant or uncontrolled extrahepatic disease, symptomatic metastatic disease, or adverse biological patterns in which treatment response must be tested without delay. Upfront ablation is defensible only when it can be completed in a single low-morbidity session and systemic therapy can begin within the interval already anticipated by the multidisciplinary treatment plan. The actual time from ablation to systemic treatment should be recorded prospectively rather than assumed to be clinically negligible.

Because no evidence-based maximum interval has been established for this specific sequence, an acceptable delay should be defined pragmatically and evaluated prospectively. After uncomplicated standalone percutaneous ablation, systemic therapy should preferably begin within 14 days and no later than the date originally specified by the MDT. Any ablation-related postponement beyond 14 days should be recorded as a protocol deviation and as a potential failure of the strategy. When ablation is performed during an otherwise indicated colorectal or hepatic operation, it should not prolong the postoperative interval to systemic therapy beyond that expected from the planned operation itself. The 14-day interval is proposed as a feasibility criterion, not as a validated oncological threshold.

Solitary versus multiple lesions. The proposal is not restricted to a solitary metastasis. It may be considered for one or a few selected lesions within multifocal disease when those lesions lie outside the planned resection field, are individually at high risk of becoming difficult to localize, and can all be ablated safely in one session without delaying systemic therapy. The remaining hepatic disease and any limited extrahepatic disease considered amenable to complete treatment must be addressed within a coherent, potentially complete, curative-intent strategy. Conversely, the concept should not be extended to indiscriminate pre-treatment ablation of numerous metastases or to patients whose dominant clinical need is immediate systemic disease control. Selection is therefore lesion-specific but must remain subordinate to the patient-level oncological plan.

Intended patient population. The most plausible candidate has liver-only disease, or exceptionally limited extrahepatic disease that is fully amenable to curative treatment; limited overall hepatic tumour burden; one or a few preferably ≤2 cm lesions outside the planned resection field; and no requirement for immediate conversion therapy. The framework may apply to synchronous or metachronous metastases, but synchronous presentation should prompt particular caution regarding occult systemic disease. It is least persuasive in high-volume, rapidly progressive, or uncontrolled extrahepatic disease, and when systemic therapy is required urgently for conversion or primarily to establish biological selection [11].

These proposed criteria should not be interpreted as defining an established clinical indication. They constitute a provisional framework for prospective investigation of a hypothesis-generating strategy and require validation before any consideration of routine clinical implementation.

## 7. Special Clinical Scenarios and Potential Liver Transplant Candidacy Percutaneous Versus Intraoperative Ablation

No single route is universally preferable. Percutaneous ablation is generally favoured for an isolated lesion that is clearly visible with ultrasonography, CT, MRI, or reliable image fusion; has a safe applicator trajectory; and can be treated with an adequate margin when no abdominal operation is otherwise planned. Its lower invasiveness and shorter recovery are particularly relevant when systemic therapy should start soon. However, planning ultrasonography may identify infeasibility because of poor lesion visibility, proximity to a large vessel, risk of collateral thermal injury to an adjacent organ, or absence of a safe access path. Laparoscopic or open intraoperative ablation may therefore be preferable when the patient is already undergoing colorectal or hepatic surgery, intraoperative ultrasonography is needed for localization, the lesion cannot be accessed safely percutaneously, adjacent bowel or another vulnerable structure requires mobilization or protection, or other hepatic lesions must be explored or treated during the same operation. The selected route should be the least invasive approach that provides reproducible targeting, adequate margins, and protection of adjacent structures.

Obstructing primary tumour requiring diversion.

An obstructing primary tumour requiring diversion or ileostomy formation may occasionally provide an opportunity for simultaneous intraoperative ablation of one or a few small CRLMs before systemic therapy. This should remain exceptional. It may be reasonable when the patient is haemodynamically stable, there is no perforation, uncontrolled sepsis, diffuse contamination, or major organ dysfunction, and the hepatic procedure is expected to add little operative time or morbidity. The lesion must be readily detectable by intraoperative ultrasonography and safely treatable with an adequate margin. In an emergency presentation with advanced obstruction, perforation, sepsis, poor physiological reserve, or an anticipated complicated postoperative course, rapid and safe control of the colorectal emergency must take precedence. Adding liver treatment in such circumstances could delay recovery and systemic therapy without an established compensating benefit. Contemporary consensus recommendations for synchronous colorectal cancer and liver metastases similarly support individualized sequencing rather than a universal combined strategy [19].

Potential candidate for liver transplantation?

Potential liver transplant candidacy should be considered as an early, separate branch of the decision framework before lesion-specific upfront ablation is selected. Liver transplantation, using either deceased-donor liver transplantation (DDLT) or living-donor liver transplantation (LDLT), is emerging as an option for highly selected patients with permanently unresectable, liver-limited CRLMs. The randomized TransMet trial evaluated transplantation plus chemotherapy in patients with a resected primary tumour, no extrahepatic disease, good performance status, BRAF-non-mutated disease, and sustained response or stability during systemic treatment. These criteria underscore that transplantation is currently relevant to a biologically selected population and not simply to any patient with technically unresectable liver disease.

Early referral to a transplant-oriented multidisciplinary team may be appropriate when liver-only unresectability is evident, particularly where an LDLT programme is available. Nevertheless, current DDLT and LDLT protocols generally require a defined period of response or stability during systemic therapy as evidence of favourable tumour biology; transplant assessment may begin early, but systemic therapy is ordinarily an essential component of selection rather than a step to be bypassed [20,21]. DDLT additionally raises questions of deceased-donor organ allocation, whereas LDLT may reduce competition for deceased-donor grafts but introduces a non-negligible risk to a healthy donor and therefore requires equally stringent oncological selection.

If total hepatectomy followed by transplantation is the intended definitive treatment, precise localization, marking, or separate ablation of every DLM is generally unnecessary because the complete native liver, including all original metastatic sites, will be removed. In this setting, disappearance may carry useful prognostic information about treatment response and tumour biology, but it does not create the same anatomical problem encountered before parenchyma-sparing resection or focal ablation. Unnecessary local treatment might instead add morbidity, alter radiological interpretation, or interfere with assessment of response. Thus, a realistic transplant pathway should usually exclude the patient from the proposed pre-treatment lesion-ablation strategy unless a specific bridge-to-transplant indication is agreed by the transplant MDT.

## 8. A Practical Research Agenda

The first research step should be a prospective multicentre feasibility study with central radiological review. Baseline hepatobiliary MRI, precise recording of lesion size and segmental location, predefined technical criteria for ablation, and documentation of the multidisciplinary rationale should all be mandatory. The study should also record whether upfront ablation delays systemic treatment, whether adequate margins are achieved, and whether the treated zone remains interpretable during follow-up.

A later comparative study could randomize patients with at least one high-risk lesion, or randomize individual suitable lesions within an overall treatment strategy, to upfront ablation followed by systemic therapy versus a contemporary standard approach. That comparator should permit fiducial placement where appropriate, because excluding this option would not reflect current precision practice. Outcomes should include lesion-level local tumour progression, unnecessary local treatment, preservation of functional liver parenchyma, completion and timing of systemic therapy, complications, need for salvage intervention, recurrence-free survival, overall survival, patient-reported burden, and cost-effectiveness.

Prospective data collection should distinguish patient-level from lesion-level variables. Patient-level variables should include primary tumour stage and sidedness, nodal status, molecular profile, synchronous or metachronous presentation, CEA and CA 19-9 kinetics, total hepatic tumour burden, extrahepatic disease status, systemic regimen, urgency of systemic treatment, and possible transplant candidacy. Lesion-level variables should include baseline diameter and conspicuity, depth, segmental location, relationship to vessels and bile ducts, planned resection field, predicted ablation margin, procedural route, early imaging response, and whether the lesion ultimately becomes radiologically occult. Recording these variables may permit development and external validation of a prediction model rather than continued reliance on unstructured MDT judgment [17,18]. Analyses should separately model radiological disappearance, failure of subsequent localization, and the management consequences of that failure; combining these outcomes would obscure the distinction central to candidate selection.

Importantly, the research agenda should not focus only on whether the ablated lesion remains controlled. It should also examine whether the strategy genuinely changes management in a beneficial way, for example, by reducing unnecessary resection of normal liver, decreasing intraoperative uncertainty, or preserving options for later salvage therapy.

## 9. Conclusions

DLMs lie at the intersection of tumour biology, advanced imaging, and liver-directed therapy. The present dilemma is not solved by assuming either that radiological disappearance equals cure or that every original site must be treated at any cost. For a carefully selected small, deep, safely ablatable CRLM that is considered at higher anticipated risk of becoming difficult to localize after systemic therapy, upfront thermal ablation is a plausible hypothesis worth formal study.

At present, this should remain an exceptional, multidisciplinary, case-based option rather than a new standard of care. Nevertheless, this question deserves careful consideration, because increasingly effective systemic therapy may cause more metastases to disappear radiologically, thereby making the management of their original sites an increasingly relevant clinical challenge. Reconsidering treatment sequence for selected lesions may therefore represent a logical next step in the broader move toward more individualized management of colorectal liver metastases.

Accordingly, the present article proposes a research question and a testable selection framework; it does not advocate routine pre-systemic-treatment ablation or modification of established CRLM pathways outside carefully documented prospective evaluation. Any prospective protocol should preserve the distinction between evidence supporting ablation as a local treatment and the still-unproven question of performing it before systemic therapy.

## Figures and Tables

**Table 1:** Potential advantages and drawbacks of upfront thermal ablation. Selected lesions at higher anticipated risk of post-treatment loss of localization.

Potential Advantages	Potential Drawbacks
Secures local treatment while the lesion remains visible and targetable	May overtreat lesions that would otherwise achieve durable complete response after systemic therapy alone
Reduces the risk of losing the lesion as an anatomical target after chemotherapy	Adds an invasive procedure before systemic therapy
May avoid empirical resection or ablation based only on historical imaging landmarks	May delay the start of urgently needed systemic treatment
May preserve healthy liver parenchyma by avoiding blind or wider-than-necessary local treatment later	May complicate interpretation of post-ablation imaging during follow-up
May simplify subsequent liver surgery when the disappearing lesion lies outside the planned resection field	Does not address the systemic biological risk of the disease
May be particularly useful for small, deep lesions that are technically straightforward before chemotherapy but difficult afterwards	Requires adequate expertise, image guidance, and the ability to obtain safe ablation margins
Preserves future options for repeat liver-directed treatment in selected patients	May remove tissue that could otherwise contribute to pathological or translational assessment
Fits a lesion-specific precision strategy in highly selected cases	Remains unsupported by prospective comparative evidence
May avoid premature interruption of neoadjuvant therapy or acceleration of planned surgery solely to treat a responding lesion before it becomes radiologically occult	Requires the risk of disappearance to be anticipated before treatment, when the subsequent response and optimal sequencing remain uncertain

## Data Availability

No new data were created or analyzed in this study. Data sharing is not applicable to this article.
